# Dynamic Susceptibility Contrast-MRI Quantification Software Tool: Development and Evaluation

**DOI:** 10.18383/j.tom.2016.00172

**Published:** 2016-12

**Authors:** Panagiotis Korfiatis, Timothy L. Kline, Zachary S. Kelm, Rickey E. Carter, Leland S. Hu, Bradley J. Erickson

**Affiliations:** 1 Department of Radiology, Mayo Clinic, Rochester, Minnesota;; 2 Department of Health Sciences Research, Mayo Clinic, Rochester, Minnesota; and; 3 Department of Radiology, Mayo Clinic, Scottsdale, Arizona

**Keywords:** dynamic susceptibility contrast, glioblastoma, atlas segmentation, white matter

## Abstract

Relative cerebral blood volume (rCBV) is a magnetic resonance imaging biomarker that is used to differentiate progression from pseudoprogression in patients with glioblastoma multiforme, the most common primary brain tumor. However, calculated rCBV depends considerably on the software used. Automating all steps required for rCBV calculation is important, as user interaction can lead to increased variability and possible inaccuracies in clinical decision-making. Here, we present an automated tool for computing rCBV from dynamic susceptibility contrast-magnetic resonance imaging that includes leakage correction. The entrance and exit bolus time points are automatically calculated using wavelet-based detection. The proposed tool is compared with 3 Food and Drug Administration-approved software packages, 1 automatic and 2 requiring user interaction, on a data set of 43 patients. We also evaluate manual and automated white matter (WM) selection for normalization of the cerebral blood volume maps. Our system showed good agreement with 2 of the 3 software packages. The intraclass correlation coefficient for all comparisons between the same software operated by different people was >0.880, except for FuncTool when operated by user 1 versus user 2. Little variability in agreement between software tools was observed when using different WM selection techniques. Our algorithm for automatic rCBV calculation with leakage correction and automated WM selection agrees well with 2 out of the 3 FDA-approved software packages.

## Introduction

Relative cerebral blood volume (rCBV) is a magnetic resonance imaging (MRI) biomarker computed from dynamic susceptibility contrast (DSC) images, and has been used extensively in brain tumor imaging for differentiation of progression versus pseudoprogression (1, 2), tumor grading (3), survival prediction (4), and tumor differentiation (5).

Perfusion analysis software to compute rCBV from DSC-MRI is widely available in clinical practice. However, it is commonly treated as a “black box,” and broad-scale integration has been slowed by the need for defining optimal methodological conditions to maximize rCBV accuracy.

The rCBV for each voxel is calculated by trapezoidal integration under the **Δ**R2*(t) area curve from the start to the end of the first-pass contrast bolus on a voxel basis divided by the value calculated for normal-appearing white matter (WM). The following pitfalls are included in processing DSC-MRI data: an inappropriate kinetic model, incorrect arterial input function (AIF) detection or deconvolution, inaccurate geometric mapping of selected anatomic regions of interest (ROIs) to the perfusion maps, incorrect calibration or normalization techniques, and incorrect identification of the bolus entrance and exit time points (6). Determining the start, end, and peak of the bolus is the most critical step of the algorithm, and it will affect calculation of percent signal recovery and mean transit time, in addition to rCBV.

DSC imaging relies on the assumptions that the gadolinium (Gd)-based contrast agent passes through tissue as a bolus and it remains within vessels without leaking into the surrounding tissue (7). Disruption of the blood–brain barrier in glioblastoma multiforme tissue violates the second assumption, and thus “leakage correction” is needed (8). Leakage correction can be addressed using one of the following methods: “preloading,” where some Gd is administered before DSC imaging (9, 10), leakage modeling during postprocessing (7, 11), by shortening the flip angle (FA) (12, 13), or with dual-echo acquisitions (14).

Although most clinical sites preload, this does not eliminate leakage. Reducing the FA decreases signal, and the use of dual-echo sequences is clinically challenging, and is currently rarely used. Thus, modeling of leakage is essential for accurate rCBV calculation. The rCBV that is calculated depends considerably on the software used (6), with modeling correction necessary for correction of residual T1 errors and T2/T2*-weighted recirculation. Currently, the method published by Boxerman et al. (11) is considered the standard for DSC-MRI leakage correction.

Recently, it was reported (15, 16) that normalization of the derived perfusion metrics to the (presumably healthy) contralateral WM resulted in increased repeatability of measurements, whereas deconvolution of the AIF may reduce repeatability. In clinical practice, a cerebral blood volume (CBV) normalized to contralateral WM without AIF deconvolution is considered to result in more repeatable values (15, 16). Currently, only 1 automated method exists for performing normalization, but it requires both WM and gray matter identification (17).

Here, we present an automated DSC-MRI quantification tool that performs leakage correction and compare it with 3 Food and Drug Administration (FDA)-approved software packages, 1 automatic and 2 requiring user interaction, on a data set of 43 patients. We also evaluate manual and automated WM selection for normalization of the CBV maps.

## Materials and Methods

### Data Set

This study was reviewed and approved by our institutional review board, with waiver of informed consent. Inclusion criteria included biopsy diagnosis of glioblastoma multiforme and treatment with radiation therapy and temozolomide administration according to Stupp protocol during the period from 2007 to 2013. In total, 43 patients where post-processing with all the 3 FDA-approved software packages was available were considered.

### MRI Protocol

Each imaging examination was acquired using a General Electric MR scanner (GE Healthcare, Milwaukee, Wisconsin), operating at 1.5 T (N = 27) or 3 T (N = 16). The T1-weighted (T1w) postcontrast images, except for 2 cases, were acquired at an oblique axial angle using either spin echo or fast-spin echo sequences ∼10 minutes after Gd injection. The T1w parameters for the 1.5 T spin echo sequence were as follows: relaxation time (TR) = 433–683 milliseconds, echo time (TE) = 20–21 milliseconds, FA = 90°, matrix = 256 × 192, field of view (FOV) = 220 × 220–250 × 250 mm, and section thickness = 4 mm, with no section gap. T1w parameters for 2-dimensional 3 T acquisitions were as follows: TR = 467–700 milliseconds, TE= 20 milliseconds, FA= 90°, matrix = 320 × 192, FOV = 220 × 220 mm, and section thickness = 4 mm, with no section gap. For both the 1.5 and 3 T scans, the DSC images were obtained using a spin echo echo-planar sequence with axial orientation and TR = 2217–2225 milliseconds, TE = 60 milliseconds, FA = 90°, matrix = 128 × 96, FOV = 240 × 240 mm, section thickness = 5 mm, and section gap = 5 mm. In total, 40 successive time points were imaged with ∼2 seconds between acquisitions. For DSC imaging, a total dose of 0.1 mmol/kg gadolinium contrast was used, with 2 cc of the dose injected about 5 minutes before preloading. The bolus injection commenced 10 seconds after acquisition started, and with transit time through the venous system, lungs, and great vessels, appearance in the brain was typically 25 seconds from start of acquisition.

### Tumor Segmentation

Enhancing tumor volumes were segmented from postsurgical, postcontrast, T1w images by a semiautomated thresholding technique. A user drew a generous boundary around the tumor, and subsequently, a thresholding technique based on Otsu thresholding was applied to finalize tumor segmentation (18).

### WM Selection

We studied both manual and automatic WM segmentation techniques. The automatic technique (automated WM selection) was designed to automatically select WM areas that were not affected by tumor, based on the DSC MRI data. To select WM areas, the DSC signal data were transformed to concentration images (19). All the data before the time point of bolus entrance were discarded. An unsupervised segmentation algorithm based on mean shift (20, 21) was used to segment the different classes of tissue based on the concentration–time curves. Because the resulting clusters did not always correspond to WM, a WM atlas was used to select the appropriate WM cluster. For this, we used the ICBM 152 Nonlinear Atlas Version 2009 (22). To register the T2-based atlas to the DSC perfusion scan, a symmetric diffeomorphic deformation model (that preserves anatomical topology even in cases requiring large deformations) was used, with mutual information as the similarity metric. The implementation was based on the ANTs registration package (23). Pixels with at least 95% WM probability were selected as the WM ROI.

For the manual WM ROI selection, two users used itk-SNAP (24) to select areas of WM contralateral to the tumor region (referred to as WM user 1 and WM user 2).

### CBV Calculation Algorithm

The following steps were used to calculate the bolus entrance and exit time points.

(1) The first 3 time points of each perfusion series are removed because of saturation effects.
(2) The average signal–time curve for the brain region is calculated.
(3) A continuous wavelet transform on the signal–time curve is performed.
(4) The maxima and minima at each scale and link across each scale are identified. The links are used to identify the maxima and minima of the curve.
(5) The signal minima and the two local maxima closest to it are located.

Subsequently, the baseline signal intensity (SI) is calculated. Next, the signal–time curves are converted to concentration–time curves based on equation 1 as follows:

(1) Δ2((t))=−ln (S(t)/Sbase)/TE

where Δ2*((t)) is the reflexivity–time curve and is a parameter related to the concentration of the Gd in a voxel, S is the dynamic SI, Sbase is the baseline, TE is the echo time, and S(t) is the dynamic SI.

CBV maps are then calculated on a voxel-wise basis using trapezoidal integration of the leakage-corrected (11) concentration–time curves between entrance and exit bolus time points recalculated for each individual pixel's corresponding concentration curve. The CBV parametric map calculation is highly affected by the selection of these points, as different choices can lead to under- or overestimation of the integration area. The rCBV map is then calculated by dividing all intensities by the mean intensity of the normal-appearing white matter CBV value.

### FDA-Approved Software Utilization

One operator created rCBV images from the DSC-MRIs using IB Neuro ver. 1.1 (Imaging Biometrics, Elm Grove, Wisconsin), as no interaction is required beyond loading the magnetic resonance images. Three operators used FuncTool ver. 4.5.3 (GE Healthcare) and nordicICE ver. 2.3.13 (NordicNeuroLab, Bergen, Norway). An effort was made to operate FuncTool and nordicICE in a similar way. FuncTool required manual selection of the prebolus baseline and integration starting and stopping time points, whereas nordicICE required manual specification of the prebolus baseline only when its automatic selection algorithm failed (7/43 cases). We used default settings for nordicICE and IB Neuro, except that leakage correction was enabled. For FuncTool, the baseline was interpolated between the integration time points. No leakage correction option was available in this version of the FuncTool.

### Statistical Methods

We compared our rCBV calculations against the 3 FDA-approved software tools' calculations using Bland–Altman analysis and the intraclass correlation coefficient (ICC). The 1-way random-effects model was used (25). The ICC reveals how strongly units in the same group resemble each other. In comparison with other correlation measures, it treats the data as groups rather than as paired observations. The analysis was performed for the tumor and the WM rCBV values.

## Results

**Figure 1.**
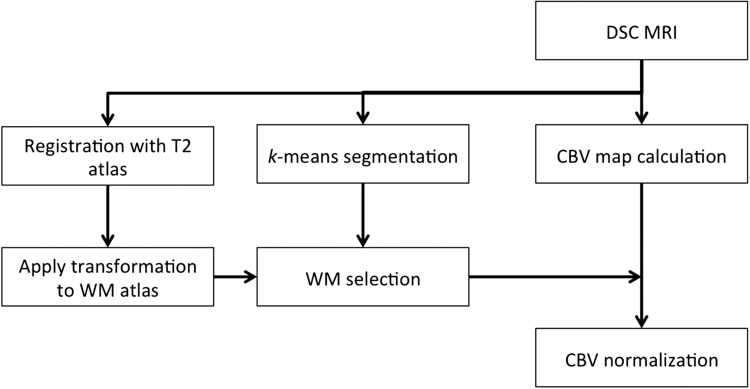
Proposed system processing pipeline.

The proposed and the FDA-approved software were used to create the CBV maps. Subsequently, the 3 ROIs available (1 automatic and 2 user-defined ROIs) were used to normalize the CBV maps. Subsequently, 2 first-order statistics, the mean value, and the 95th percentile, were calculated for each patient of the data set. Figure 1 illustrates the processing pipeline of the proposed system.

**Figure 2.**
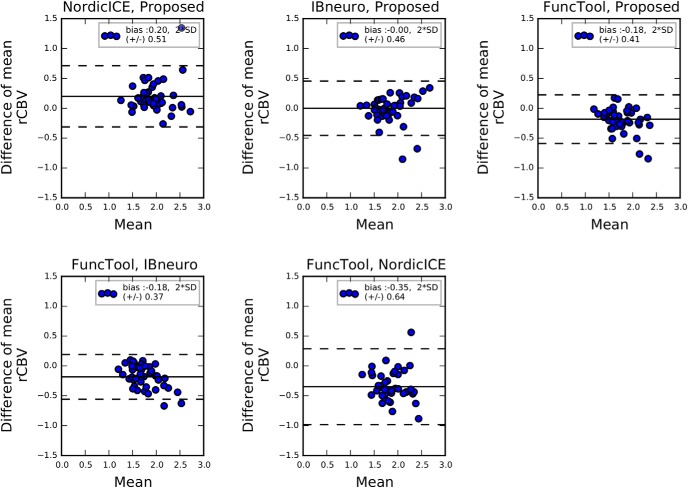
Bland–Altman plots for the mean relative cerebral blood volume (rCBV) values between the Food and Drug Administration (FDA)-approved and the proposed software systems for the mean rCBV measurement. The solid line represents the mean value for the data points and the slashed line represents the 2*SD.

**Figure 3.**
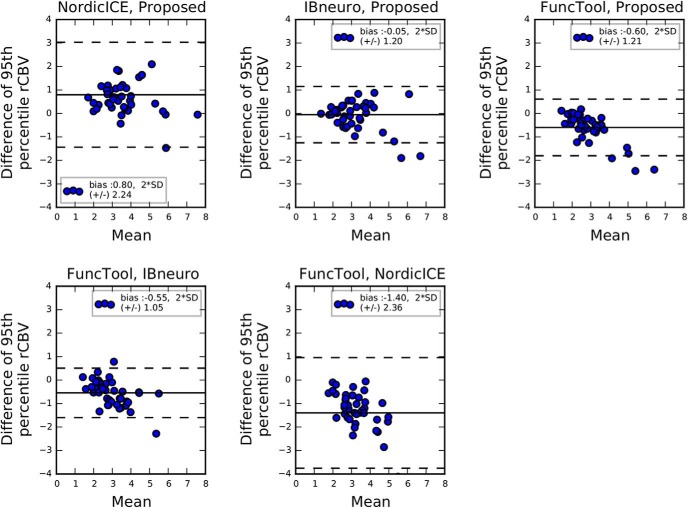
Bland–Altman plots for the 95th percentile rCBV values between the FDA-approved and the proposed software systems for the 95th percentile rCBV measurement. The solid line represents the mean value for the data points and the dashed line represents the 2*SD.

The proposed system shows excellent agreement with 2 of the 3 FDA-approved software considered in this study. Figures 2 and 3 are the Bland–Altman plots for the difference in measured values between the FDA-approved and the proposed system for the mean and 95th percentile of rCBV, respectively. Based on the plots, the proposed system falls within 2 standard deviations (SDs) of the FDA-approved software systems, which is similar to the agreement between the 2 FDA cleared systems. A larger bias was observed in case of NordicICE compared with our system (0.20 and 1.34 for mean rCBV and 95th percentile rCBV, respectively).

Figures 2 and 3 reveal the difference between the mean and the 95th percentile rCBV in the sensitivity of the imaging biomarkers, as 95th percentile reflects only a very small number of pixels, and thus is more sensitive to local artifacts. Smoothing or low-pass filtering will also affect this measure.

**Figure 4.**
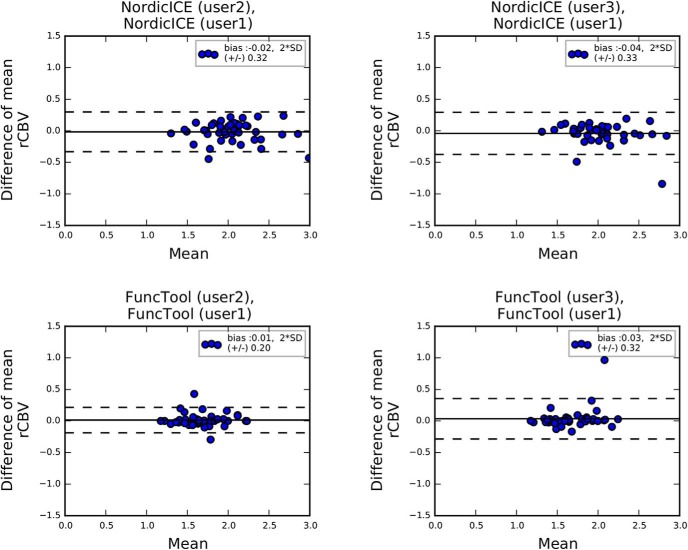
Bland–Altman plots for the mean rCBV values between FDA-approved software requiring user input for the mean rCBV measurement. The solid line represents the mean value for the data points and the dashed line represents the 2*SD.

**Figure 5.**
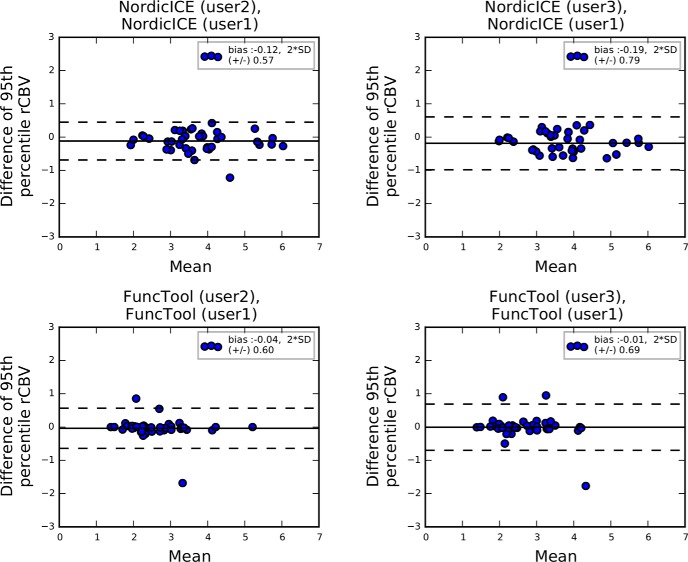
Bland–Altman plots for the 95th percentile rCBV values between FDA-approved software requiring user input for the 95th percentile rCBV measurement. The solid line represents the mean value for the data points and the dashed line represents the 2*SD.

Figures 4 and 5 correspond to Bland–Altman plots for the difference of measured values between the two packages requiring user interaction (mean and 95th rCBV, respectively). In all cases for the mean rCBV, the bias was <0.03. The largest 2*SD observed was 0.41 in the case of FuncTool for users 1 and 2. This range is comparable with the range observed between the proposed system and the 3 FDA-approved software. The 95th rCBV and the 2*SD are lower than those observed for the software comparison in all cases.

**Figure 6.**
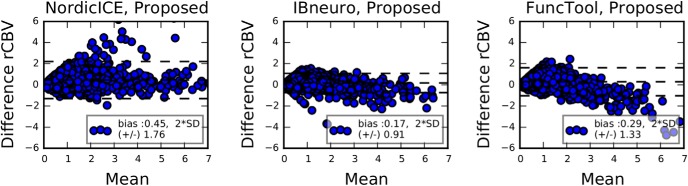
Bland–Altman plots for rCBV values corresponding to a specific tumor between all FDA-approved software systems with different user setup and the proposed system. The solid line represents the mean value for the data points and the dashed line represents the 2*SD.

**Figure 7.**
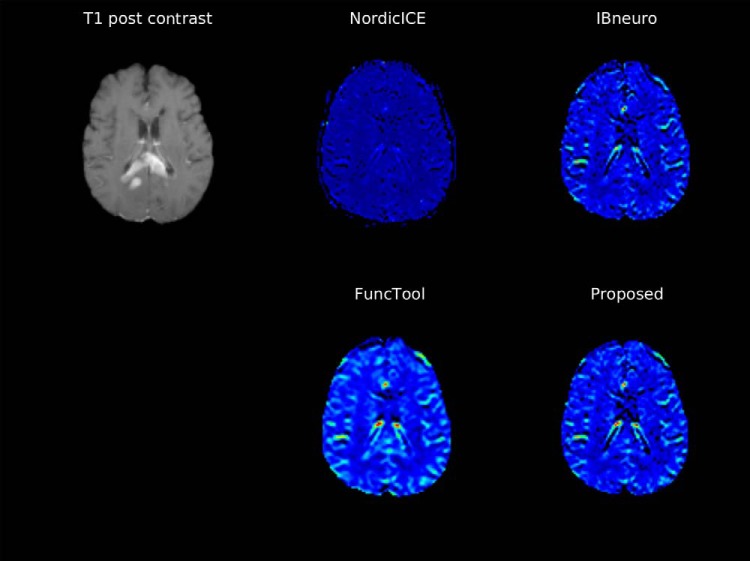
Cerebral blood volume (CBV) maps created with the software tools used in this study and the corresponding post-contrast T1 image.

**Figure 8.**
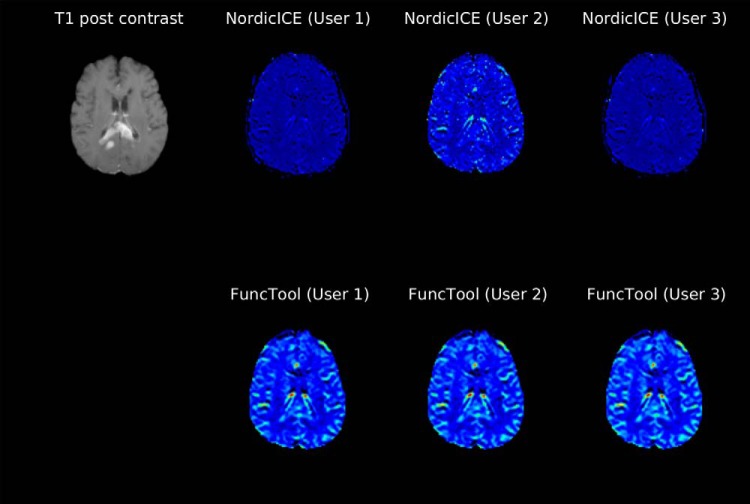
CBV maps created with NordicICE (row 1) and FuncTool (row 2) produced by the 3 operators participating in this study.

Figure 6 displays the Bland–Altman plots for rCBV values corresponding to the area of a tumor selected from our data set for all software tools considered in this study. Proportional bias is observed between FuncTool and the proposed method. The existence of proportional bias indicates that the methods do not agree equally through the range of measurements. This can be attributed to the smoothing observed in the CBV maps created by FuncTool (Figure 7). Figures 7 and 8 depict the CBV maps created by the 4 software tools considered in this study for 2 different subjects.

Table 1 summarizes the ICC for both mean and 95th percentile rCBV for all the comparisons considered in this study and all the methods for normalization used in this study. The results from the proposed system correlate better with IBNeuro and FuncTool. FuncTool results are less correlated with IBNeuro, whereas ICC is good between FuncTool and IBNeuro. The ICC for all the comparisons considered between the same software operated by different people was >0.880 with the exception of FuncTool when operated by user 1 versus user 2.

**Table 1.** ICC for the 3 Software and Our Tool

ROI	Comparisons	Tumor–ICC (95^th^ percentile rCBV)	Tumor–ICC (mean rCBV)	WM–ICC (95^th^ percentile rCBV)	WM–ICC (mean rCBV)
Automated WM selection	NordicICE vs proposed	0.664	0.770	0.754	0.800
	IBneuro vs proposed	0.868	0.780	0.881	0.823
	FuncTool vs proposed	0.803	0.810	0.795	0.845
	NordicICE vs IBneuro	0.572	0.680	0.657	0.699
	FuncTool vs IBneuro	0.700	0.670	0.745	0.779
	NordicICE (user 1) vs NordicICE (user 2)	0.977	0.900	0.77	0.824
	NordicICE (user 1) vs NordicICE (user 3)	0.949	0.880	0.784	0.794
	FuncTool (user 1) vs FuncTool (user 2)	0.922	0.920	0.754	0.806
	FuncTool (user 1) vs FuncTool (user 3)	0.893	0.830	0.815	0.927
	NordicICE vs FuncTool	0.156	0.160	0.767	0.864
WM user 1	NordicICE vs proposed	0.57	0.515	0.952	0.908
	IBneuro vs PROPOSED	0.745	0.821	0.732	0.632
	FuncTool vs proposed	0.759	0.804	0.933	0.921
	NordicICE vs IBneuro	0.353	0.539	0.743	0.771
	FuncTool vs IBneuro	0.744	0.757	0.738	0.708
	NordicICE (user 1) vs NordicICE (user 2)	0.951	0.939	0.745	0.769
	NordicICE (user 1) vs NordicICE (user 3)	0.958	0.917	0.71	0.83
	FuncTool (user 1) vs FuncTool (user 2)	0.932	0.834	0.79	0.807
	FuncTool (user 1) vs FuncTool (user 3)	0.895	0.894	0.985	0.988
	NordicICE vs FuncTool	0.17	0.305	0.936	0.928
WM user 2	NordicICE vs proposed	0.569	0.606	0.823	0.742
	IBneuro vs proposed	0.754	0.814	0.773	0.605
	FuncTool vs proposed	0.782	0.822	0.787	0.747
	NordicICE vs IBneuro	0.401	0.674	0.767	0.645
	FuncTool vs IBneuro	0.743	0.804	0.685	0.624
	NordicICE (user 1) vs NordicICE (user 2)	0.949	0.941	0.846	0.852
	NordicICE (user 1) vs NordicICE (user 3)	0.944	0.912	0.716	0.836
	FuncTool (user 1) vs FuncTool (user 2)	0.724	0.639	0.877	0.854
	FuncTool (user 1) vs FuncTool (user 3)	0.717	0.697	0.99	0.972
	NordicICE vs FuncTool	0.018	0.161	0.863	0.787

Abbreviations: ROI, regions of interest; ICC, intraclass correlation coefficient; WM, white matter; rCBV, relative cerebral blood volume.

Little variability in agreement between software tools was observed when using different WM selection techniques. All software tools yielded high ICC for both the 95th and mean rCBV metrics for both automated and manual WM selection techniques.

## Discussion

Here, we present a method for estimating rCBV metrics from DSC-MRI with an automated WM selection step using a probabilistic atlas to further standardize rCBV calculation. This automated technique was found to be in close agreement with manual WM selection.

Our software is in good agreement with IBNeuro and FuncTool for tumor rCBV measurements of both mean and the 95th percentile values based on ICC. Mean rCBV ICC values are higher than the 95th percentile measures, most likely because the 95th percentile value is more sensitive to outliers, as it reflects a much smaller sample size.

User interaction can lead to increased variation in the rCBV measurements (Figures 4 and 5), which is more pronounced in the case of NordicICE.

We compared both the rCBV metrics originating from tumor regions and from WM ROIs, both manually and by automatic selection. The ICC for measures extracted from the tumor ROI for each software comparison for the different WM selection methods was similar, revealing good agreement in the majority of the comparisons. This was not observed when using the ROI selections made by user 2.

Calculation of rCBV for the tumor region is expected to be more complicated because of leakage effects. However, based on our analysis, it seems that WM rCBV values vary by software package. Selection of the normalizing WM ROI is crucial, as it can lead to further increases in the variation of rCBV measurements.

Further investigation is needed with respect to the variation introduced because of the software used for rCBV calculation and the clinical impact. Recently, Kelm et al. (6) showed that the software used for rCBV calculation can result in very different values, and that across a range of clinically relevant thresholds, clinical decisions will be different in a large fraction of patients. Hu et al. (7), recently, compared IBNeuro and NordicICE modeling methods in a cohort of 52 patients with glioma. They found that IBNeuro showed significantly better T1w leakage correction compared with NordicICE. Similarly, rCBV, as measured on IBNeuro, showed stronger correlation with image-guided microvessel quantification and higher accuracy in predicting tumor recurrence from pseudoprogression/radiation necrosis, based on validation by surgical histologic tumor quantification. Orsingher et al. (26) also found significant differences in rCBV values when comparing General Electric and NordicICE software packages. The findings of both these studies coincide with the findings of the current study.

One of the limiting factors of this study is the fact that proprietary commercial packages were used and thus many details of the algorithmic implementation were unavailable for explaining the differences observed. The variation in measurements seen is likely due to differences in how the DSC-MRI intensities are modeled. Other limitations of this study include the use of spin echo technique with less than a single-dose bolus injection and low amounts of preload dose. Gradient-echo T2*-weighted DSC represents the more commonly used and widely published method for DSC. Although spin echo T2-weighted DSC offers a higher signal-to-noise ratio and fewer susceptibility artifacts, double or triple doses of contrast bolus may be required to achieve sufficient bolus response in every case (7).

In addition, the images used for this work were obtained on just one vendor platform (a limitation imposed by one of the software packages we compared). However, we expect that including more scanner vendor platforms would likely increase the variance between software.

The proposed algorithm and the analysis was performed using Python (Python, Python Software Foundation: v.2.7.4, numpy: v.1.8.1, scipy: v.0.13.3, matplotlib: v1.5.0).

## Conclusion

We describe an automatic algorithm for rCBV calculation that includes leakage correction and an automated step for WM selection. The algorithm was evaluated against 3 FDA-approved software packages with respect to tumor and WM ROIs. The proposed system has good agreement with 2 out of 3 software tools considered.
